# Loss of lysosomal membrane protein NCU-G1 in mice results in spontaneous liver fibrosis with accumulation of lipofuscin and iron in Kupffer cells

**DOI:** 10.1242/dmm.014050

**Published:** 2014-01-30

**Authors:** Xiang Y. Kong, Cecilie Kasi Nesset, Markus Damme, Else-Marit Løberg, Torben Lübke, Jan Mæhlen, Kristin B. Andersson, Petra I. Lorenzo, Norbert Roos, G. Hege Thoresen, Arild C. Rustan, Eili T. Kase, Winnie Eskild

**Affiliations:** 1 Department of Bioscience, University of Oslo, 0316 Oslo, Norway.; 2 Institut für Biochemie, Christian-Albrechts-Universität zu Kiel, Olshausenstrasse 40, 24098 Kiel, Germany.; 3 Department of Biochemistry I, Bielefeld University, 33615 Bielefeld, Germany.; 4 Department of Neuroradiology, Oslo University Hospital Ullevaal, Kirkeveien 166, NO-0450 Oslo, Norway.; 5 Institute for Experimental Medical Research, Oslo University Hospital Ullevaal, Kirkeveien 166, NO-0450 Oslo, Norway.; 6 Department of Pharmaceutical Biosciences, School of Pharmacy, University of Oslo, Blindern, 0316 Oslo, Norway.; 7 Department of Pharmacology, Institute of Clinical Medicine, Faculty of Medicine, University of Oslo and Oslo University Hospital, Problemveien 7, 0313 Oslo, Norway.

**Keywords:** NCU-G1, Lysosome, Fibrosis

## Abstract

Human kidney predominant protein, NCU-G1, is a highly conserved protein with an unknown biological function. Initially described as a nuclear protein, it was later shown to be a bona fide lysosomal integral membrane protein. To gain insight into the physiological function of NCU-G1, mice with no detectable expression of this gene were created using a gene-trap strategy, and *Ncu-g1^gt/gt^* mice were successfully characterized. Lysosomal disorders are mainly caused by lack of or malfunctioning of proteins in the endosomal-lysosomal pathway. The clinical symptoms vary, but often include liver dysfunction. Persistent liver damage activates fibrogenesis and, if unremedied, eventually leads to liver fibrosis/cirrhosis and death. We demonstrate that the disruption of *Ncu-g1* results in spontaneous liver fibrosis in mice as the predominant phenotype. Evidence for an increased rate of hepatic cell death, oxidative stress and active fibrogenesis were detected in *Ncu-g1^gt/gt^* liver. In addition to collagen deposition, microscopic examination of liver sections revealed accumulation of autofluorescent lipofuscin and iron in *Ncu-g1^gt/gt^* Kupffer cells. Because only a few transgenic mouse models have been identified with chronic liver injury and spontaneous liver fibrosis development, we propose that the *Ncu-g1^gt/gt^* mouse could be a valuable new tool in the development of novel treatments for the attenuation of fibrosis due to chronic liver damage.

## INTRODUCTION

Human kidney predominant protein, NCU-G1, is a protein whose function is only beginning to emerge. First reported in 1994 (Eskild et al., 1994), mouse NCU-G1 was cloned in 2001 (Kawamura et al., 2001). Initial studies described NCU-G1 as a nuclear protein with gene regulatory properties (Steffensen et al., 2007), whereas more recent reports have shown that NCU-G1 is a bona fide lysosomal type I integral membrane protein (Sardiello et al., 2009; Schieweck et al., 2009; Schröder et al., 2010). NCU-G1 is highly conserved, proline-rich and ubiquitously expressed; however, it shows no sequence homology to other known proteins.

The endosomal-lysosomal pathway is sensitive to protein dysfunctions, and alterations in protein composition and function can lead to a group of metabolic diseases categorized as lysosomal disorders (Futerman and van Meer, 2004; Parkinson-Lawrence et al., 2010). Intralysosomal accumulation of unmetabolized substrates can result from malfunction of any endosomal-lysosomal protein, but the severity and clinical manifestations vary owing to secondary alterations in other biochemical and cellular pathways (Futerman and van Meer, 2004), such as apoptosis and elevated levels of reactive oxygen species due to impaired lysosomal clearance of damaged organelles through autophagy (Tardy et al., 2004; Kiselyov et al., 2007; Kurz et al., 2008; Cox and Cachón-González, 2012). Certain cell types are more sensitive to lysosomal dysfunction than others, especially cells with low proliferation capacity (Kiselyov et al., 2007; Kurz et al., 2008; Cox and Cachón-González, 2012). This is reflected in the vast frequency of neurological symptoms in different lysosomal disorders (Parkinson-Lawrence et al., 2010; Cox and Cachón-González, 2012; Platt et al., 2012). For some disorders, however, other symptoms like liver damage are also a consistent clinical finding (Platt et al., 2012).

Persistent injury to the liver eventually leads to liver fibrosis and deposition of excess connective tissue (Bataller and Brenner, 2005; Friedman, 2008; Gieling et al., 2008; Kisseleva and Brenner, 2011; Ramachandran and Iredale, 2012). The development of liver fibrosis involves several liver cell types and immune cells, including hepatocytes, hepatic stellate cells (HSCs), Kupffer cells (KCs), endothelial cells and infiltrating leukocytes (Friedman, 2008; Pinzani and Macias-Barragan, 2010; Hernandez-Gea and Friedman, 2011). Dying cells secrete reactive oxygen species, and release necrotic and apoptotic fragments, leading to activation of KCs and recruitment of inflammatory cells to the site of injury (Bataller and Brenner, 2005; Friedman, 2008). Activated KCs and recruited leukocytes contribute to the activation of HSCs into myofibroblast-like cells by secreting pro-fibrogenic cytokines, including transforming growth factor-β (TGF-β) and platelet-derived growth factor (PDGF) (Bataller and Brenner, 2005; Friedman, 2008). When activated, HSCs start secreting high amounts of extracellular matrix (ECM) proteins that differ from those normally expressed, including fibrillar collagen, thus replacing dead parenchymal cells with fibrous ECM, ultimately compromising the organ function if untreated (Gressner et al., 2007; Mormone et al., 2011).

**TRANSLATIONAL IMPACT**

**Clinical issue**

Lysosomal storage disorders are a group of inherited metabolic disorders that are caused by malfunction of the endosomal-lysosomal pathway, often with devastating consequences for patients. Most lysosomal disorders exhibit neurological symptoms, but internal organ injury and liver damage are consistent clinical findings in several lysosomal disorders. Indeed, recent research has uncovered a direct link between abnormal lysosomal function and liver fibrosis – scarring of the liver in response to chronic injury that eventually leads to liver cirrhosis. Currently, due to a shortage of more suitable models, the development of liver fibrosis is often studied in animal models that involve experimental induction of liver damage, which leads to acute fibrosis. An animal model of chronic fibrosis is needed to help improve our understanding of this condition.

**Results**

Transgenic mouse models of lysosomal disorders are valuable tools for characterization of the molecular mechanisms behind the broad spectrum of clinical symptoms, including liver fibrosis. In this study, the authors develop a novel mouse model with no detectable expression of the lysosomal membrane protein, NCU-G1, using a gene-trap strategy; NCU-G1 is highly expressed in liver, kidney, lung and prostate and might be crucial for normal lysosomal function. The authors report that disruption of *Ncu-g1* in mice does not interfere with fertility, normal embryonic development or growth. They show that the predominant phenotype of *Ncu-g1^gt/gt^* mice is spontaneous development of liver fibrosis, accompanied by splenomegaly. Using gene expression analyses, the authors show that *Ncu-g1^gt/gt^* mice exhibit all the hallmarks of well-established liver fibrosis by the age of 6 months. Finally, they report that NCU-G1 deficiency leads to accumulation of lipofuscin and iron in Kupffer cells in the liver.

**Implications and future directions**

These results indicate that disruption of NCU-G1 function leads to chronic liver injury and activation of fibrogenesis in mice. *Ncu-g1^gt/gt^* mice therefore represent a potential animal model for studies of these conditions and for the development of treatments for liver fibrosis. Further studies using this model will enable a more extensive characterization of the biochemical and molecular pathways that are affected during chronic liver damage and fibrogenesis, and will determine whether abnormal storage of lipofuscin and iron in Kupffer cells is a primary or secondary effect of NCU-G1 ablation. Finally, these findings suggest that disruption of *Ncu-g1* might be causative for a previously undescribed lysosomal disorder that has liver fibrosis as its predominant phenotype.

Several rodent models for inducible liver fibrosis are available (Weiler-Normann et al., 2007; Starkel and Leclercq, 2011), but only a few transgenic mouse models have been identified that develop spontaneous liver fibrosis (Fickert et al., 2002; Takehara et al., 2004; Wandzioch et al., 2004; Warskulat et al., 2006). Mouse models are widely used in the study of lysosomal disorders and the impact of lysosomal protein deficiency (Parkinson-Lawrence et al., 2010; Platt et al., 2012). However, the physiological consequences of loss of NCU-G1 function have never been characterized before. The lysosomal localization suggests some alterations in the endosomal-lysosomal pathway following *Ncu-g1* gene disruption. In this article, we present a new mouse model, created by using a gene-trap strategy to introduce a transcriptional stop codon to intron 1 of the *Ncu-g1* gene (*Ncu-g1^gt/gt^*). *Ncu-g1^gt/gt^* mice have no detectable expression of NCU-G1, display normal growth and fertility, and have a life expectancy up to at least 18 months. The predominant phenotype is a spontaneous development of liver fibrosis by the age of 6 months. Analyses of *Ncu-g1^gt/gt^* liver indicate that absence of this lysosomal membrane protein leads to an increased rate of hepatic cell death, oxidative stress, activation of the fibrogenic response, and accumulation of lipofuscin and iron in KCs.

## RESULTS

### Successful generation of *Ncu-g1^gt/gt^* mice

**Fig. 1.**
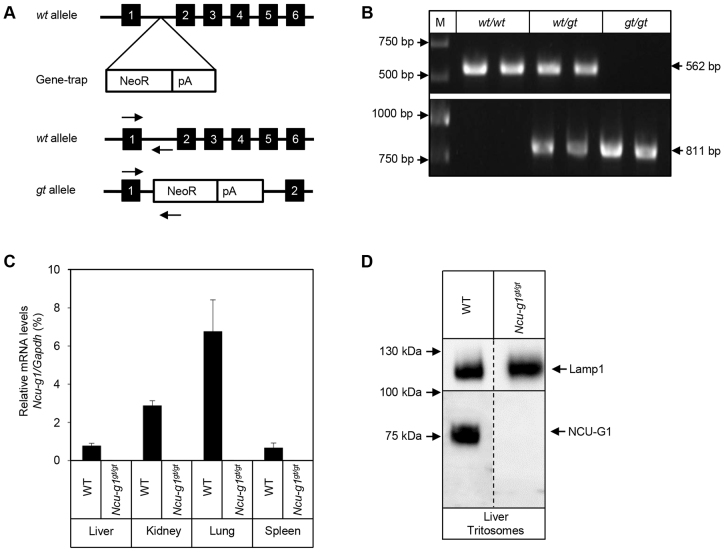
**Verification of *Ncu-g1* gene disruption.** (A) Schematic representation of gene-trap vector insertion into the *Ncu-g1* locus (top). The wild-type (*wt*) allele (middle) and the trapped (*gt*) allele (bottom) are shown. The targeting vector, consisting of a neomycin-resistance cassette (NeoR) and a polyadenylation site (pA), was inserted into intron 1 of the *wt* allele to produce a gene-trap (*gt*) allele. Arrows indicate the hybridization sites for genotyping primers (*NCUG1* and *FlpROSA*, for *wt* and *gt* alleles, respectively). PCR products of 562 bp indicate the presence of *Ncu-g1 wt* allele and 811 bp the *gt* allele, respectively. (B) Genotyping of mice by PCR. The *Ncu-g1 wt* 562 bp PCR product was present in both homozygous wild-type (*wt/wt*) and heterozygous (*wt/gt*) samples, but not in homozygous gene-trap (*gt/gt*) samples. Conversely, the 811 bp gene-trap PCR product was present in *gt/gt* and *wt/gt* samples, but not in *wt/wt* samples. (C) The absence of mRNA transcripts in *Ncu-g1^gt/gt^* was verified in liver, kidney, lung and spleen, using TaqMan qPCR and *Gapdh* as a reference gene. (D) No NCU-G1 protein was detected by western blot analyses of *Ncu-g1^gt/gt^* liver tritosomes. Lamp1 served as a loading control.

Breeding of heterozygous mice carrying a gene-trap cassette inserted into intron 1 of the *Ncu-g1* gene (Fig. 1A) resulted in three expected genotypes (*Ncu-g1^wt/wt^, Ncu-g1^wt/gt^* and *Ncu-g1^gt/gt^*) (Fig. 1B). Expression of mRNA for *Ncu-g1* was analyzed to assess any transcription leakage from the gene-trap. Fig. 1C shows a total elimination of *Ncu-g1* mRNA in homozygous gene-trap mice compared with wild-type mice in all organs studied. Lysosome-enriched fractions from mouse liver after tyloxapol treatment (Schieweck et al., 2009) were used to confirm the loss of NCU-G1 at the protein level as shown in Fig. 1D. Genotype and sex distributions for a total of 364 pups generated by four heterozygous breeding pairs were analyzed and found to be in accordance with Mendelian distributions (*P*<0.001), indicating that NCU-G1 was not essential for mouse embryonic development (data not shown). Heterozygotes and homozygotes had similar growth rates as their wild-type siblings. When mated, homozygous *Ncu-g1^gt/gt^* mice produced litters at the same frequency and of a similar litter size as wild-type and heterozygote mating (data not shown).

### Phenotype of *Ncu-g1^gt/gt^* mice

**Fig. 2.**
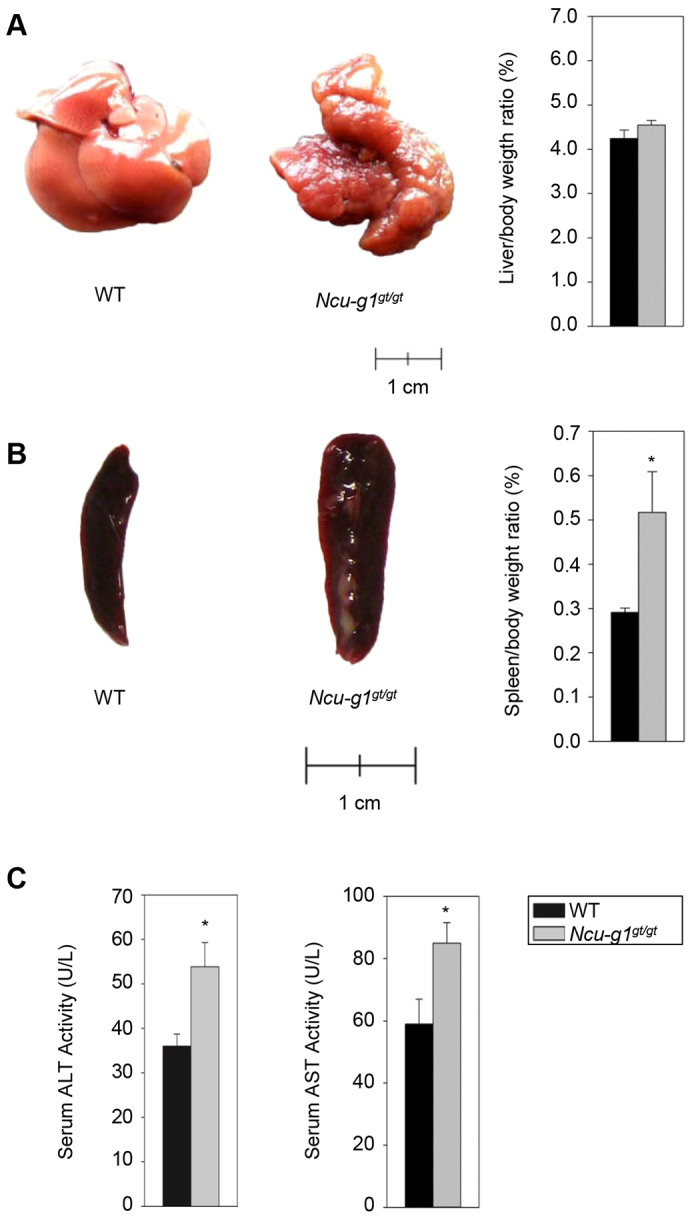
**Loss of NCU-G1 leads to liver fibrosis and splenomegaly in 6-month-old mice.** (A) Representative livers from wild-type (WT) and *Ncu-g1^gt/gt^* mice. Wild-type liver has a smooth surface with easily recognizable individual lobes, whereas the *Ncu-g1^gt/gt^* liver has a fibrotic appearance and a contracted, distorted and nodular appearance. Disruption of *Ncu-g1* did not result in hepatomegaly, as indicated by the liver weight:body weight ratio (*P*=0.23, *n*=6–7). (B) Representative pictures of a wild-type (WT) and *Ncu-g1^gt/gt^* spleen. Spleen weight:body weight ratio of WT and *Ncu-g1^gt/gt^* spleens shows a significantly enlarged spleen in *Ncu-g1^gt/gt^* mice (*n*=6–7, *P=0.02). (C) Serum ALT and AST activity levels are significantly elevated in *Ncu-g1^gt/gt^* mice (*n*=4, *P<0.05). Values are expressed as mean±s.e.m.

At birth, *Ncu-g1^gt/gt^* mice were indistinguishable from wild-type siblings, and displayed no obvious behavioral differences. At the age of 6 months, lack of NCU-G1 resulted in a fibrotic appearance of the liver, with large nodules on the surface (Fig. 2A). However, the liver:body weight ratio was unaffected (Fig. 2A). Splenomegaly was observed in *Ncu-g1^gt/gt^* mice with a nearly doubled spleen:body weight ratio (Fig. 2B). This is probably a result of portal vein hypertension caused by the liver fibrosis. Measurements of serum alanine aminotransferase (ALT) and aspartate aminotransferase (AST) activities showed moderate but significant increases in *Ncu-g1^gt/gt^* mice compared with wild-type animals (Fig. 2C). Other organs (kidney, lung, heart, brain) appeared normal and were not further evaluated in this study. Interestingly, *Ncu-g1^gt/gt^* mice were kept until the age of 18 months before termination, indicating that the liver fibrosis does not progress to a lethal condition during this period.

### Inflammation and excess collagen in *Ncu-g1^gt/gt^* liver

In order to gain insight into the underlying mechanisms behind the *Ncu-g1^gt/gt^* mouse phenotype, qPCR arrays were used to identify changes in gene expression in groups of genes known to be involved in liver fibrogenesis. This initial screening suggested that genes associated with fibrosis, hepatotoxicity and oxidative stress responses were regulated in *Ncu-g1^gt/gt^* liver (supplementary material Table S1). In addition, a screening of different signal transduction pathways suggested a strong upregulation of genes encoding chemokines and proteins involved in control of the cell cycle (supplementary material Table S1). A selection of these genes was verified by qPCR (see below).

**Fig. 3.**
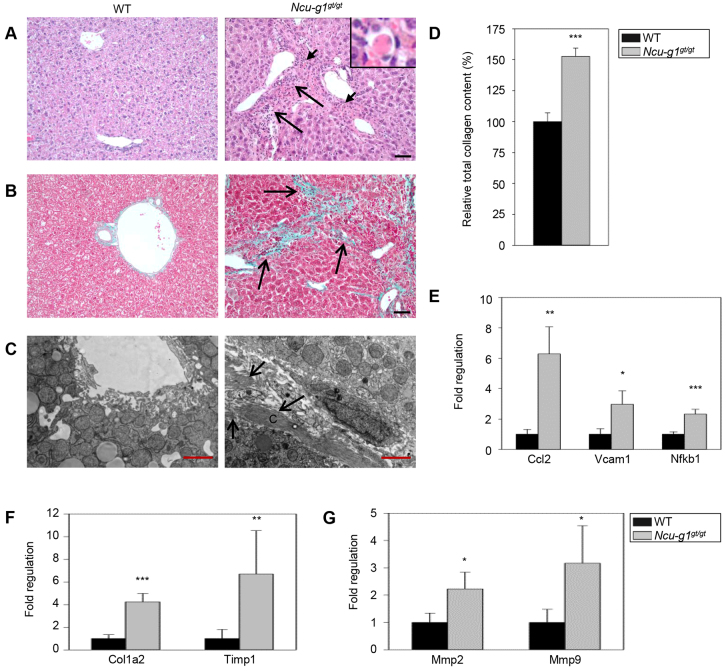
**Inflammation, dying hepatocytes and collagen deposition in *Ncu-g1^gt/gt^* liver.** (A) Hematoxylin and eosin staining of liver sections from 6-month-old wild-type (WT) and *Ncu-g1^gt/gt^* mice with a distorted tissue structure, massive infiltration of mononuclear leucocytes and granulocytes (long arrows), and acidophilic hepatocytes (short arrows, inset) in portal areas of *Ncu-g1^gt/gt^* mice. Scale bar: 50 μm. (B) Masson-Goldner’s trichrome staining of liver sections from WT and *Ncu-g1^gt/gt^* mice shows fibrosis in periportal regions of *Ncu-g1^gt/gt^* mice (arrows), where connective tissue stains green. Scale bar: 50 μm. (C) Representative transmission electron microscopy micrographs of perisinusoidal areas in liver sections from WT and *Ncu-g1^gt/gt^* mice. Massive deposition of fibrous collagen is present in the space of Disse of the *Ncu-g1^gt/gt^* liver (arrows) (C, collagen). Scale bars: 2 μm. (D) Relative total collagen content in WT and *Ncu-g1^gt/gt^* liver as measured by sirius-red colorimetric plate assay. (E) qPCR analyses show elevated expression of genes involved in leukocyte recruitment (*Ccl2, Vcam1, Nfkb1*), and (F,G) extracellular matrix remodeling (*Col1a1, Timp1, Mmp2, Mmp9*) in *Ncu-g1^gt/gt^* liver (*n*=4, *P<0.05, **P<0.005, ***P<0.001). Values are expressed as mean±s.e.m.

Light-microscopic examination of hematoxylin and eosin (H&E)-stained liver sections from *Ncu-g1^gt/gt^* mice showed infiltration of mononuclear leukocytes and granulocytes in the portal areas with some piecemeal necrosis (Fig. 3A). Widespread deposition of collagen was revealed in sections stained with Masson-Goldner’s trichrome (Fig. 3B). This observation was supported by electron microscopy, showing thick collagen fibers in the space of Disse (Fig. 3C) and sirius-red quantification of total collagen content in liver homogenates (Fig. 3D). In affected areas, loss of hepatocytes and bridging fibrosis resulted in disruption of the normal tissue architecture (Fig. 3A,C). Expression of genes such as *Vcam1, Ccl2* and *Nfkb1*, involved in leukocyte recruitment (Fig. 3E), and *Mmp2, Mmp9, Timp1* and *Col1a2*, involved in ECM remodeling (Fig. 3F,G), were found to be elevated in *Ncu-g1^gt/gt^* liver, as detected by qPCR analysis, supporting the microscopy findings.

### Increased rate of cell death and oxidative stress in *Ncu-g1^gt/gt^* liver

**Fig. 4.**
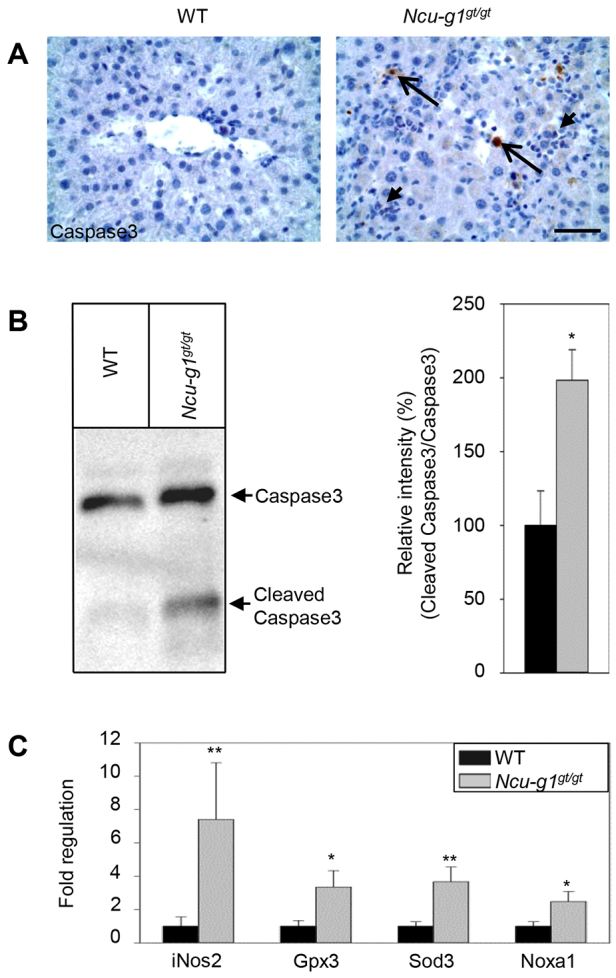
**Increased rate of cell death and oxidative stress in *Ncu-g1^gt/gt^* liver.** (A) Liver sections labeled for active caspase 3 and counterstained with Mayer’s hematoxylin indicate apoptosis (long arrows) and inflammation (short arrows) in *Ncu-g1^gt/gt^* mice. Scale bar: 50 μm. (B) Representative western blot showing caspase 3 expression in wild-type (WT) and *Ncu-g1^gt/gt^* liver. The levels of active caspase 3 were significantly higher in *Ncu-g1^gt/gt^* liver homogenates (*n*=4, *P<0.05). (C) Expression of genes involved in oxidative stress (*iNos2, Gpx3, Sod3, Noxa1*) were elevated in *Ncu-g1^gt/gt^* liver (*n*=4, *P<0.05, **P<0.005). Values are expressed as mean±s.e.m.

In the *Ncu-g1^gt/gt^* livers, scattered acidophilic hepatocytes were seen (Fig. 3A, insert). Immunoreactivity for active caspase 3, a specific marker for apoptosis, showed an increased number of apoptotic cells in *Ncu-g1^gt/gt^* mouse liver (Fig. 4A). Increased levels of active caspase 3 were also confirmed by western blot (Fig. 4B). Leukocyte infiltration and damage to hepatocytes are often associated with increased oxidative stress (Friedman, 2008), as already indicated by qPCR array analyses. Fig. 4C shows the increased levels of expression of a selection of genes associated with oxidative stress: *iNos2, Gpx3, Sod3* and *Noxa1*.

### Active fibrogenesis in *Ncu-g1^gt/gt^* liver

**Fig. 5.**
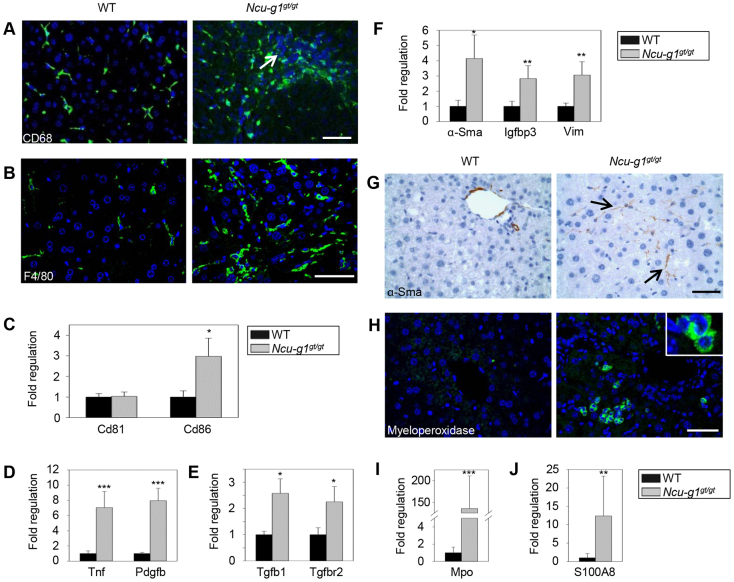
**Activated fibrogenic response in *Ncu-g1^gt/gt^* liver.** (A,B) Immunofluorescence image shows CD68- and F4/80-stained KCs (green). Liver sections were counterstained with DAPI (blue). Infiltrating leukocytes can be seen in *Ncu-g1^gt/gt^* liver section (white arrow). CD68-positive cells seem to congregate at the site of leukocyte infiltration. In *Ncu-g1^gt/gt^* liver sections, F4/80-positive cells appear swollen and hypertrophic. Scale bars: 50 μm. (C) qPCR analyses of the restorative (*Cd81*) and the pro-inflammatory (*Cd86*) macrophage markers show elevated expression of only *Cd86* in *Ncu-g1^gt/gt^* liver. (D,E) Activated KCs produce pro-fibrogenic cytokines. qPCR analyses show elevated expression of genes characteristic for activated fibrogenesis (*Tnf, Pdgfb, Tgfb1, Tgfbr2*). (F) Presence of activated HSCs was indicated by increased expression of genes characteristic for activated HSCs (*α-Sma, Igfbp3, Vim*). (G) This was supported by α-Sma staining in liver sections from wild-type (WT) and *Ncu-g1^gt/gt^* mice. Note the positive labeling of α-Sma indicating activated HSCs (arrows) in affected areas. Liver sections were counterstained with hematoxylin. Scale bar: 50 μm. (H) Immunofluorescence imaging shows increased levels of myeloperoxidase at the protein level in *Ncu-g1^gt/gt^* liver (green). Liver sections were counterstained with DAPI (blue). Note the granular staining pattern of myeloperoxidase, clustering of myeloperoxidase-positive cells and increased levels of invading polymorphonuclear leukocytes with small nuclei. Scale bar: 50 μm. (I) qPCR analyses supported the elevated myeloperoxidase (*Mpo*) expression in *Ncu-g1^gt/gt^* liver. (J) The expression of S100 calcium binding protein A8 (*S100a8*) was also elevated in *Ncu-g1^gt/gt^* liver. (C-F,I,J) *n*=4, *P<0.05, **P<0.005, ***P<0.001. Values are expressed as mean±s.e.m.

Injury to hepatic cells and oxidative stress are inducers of liver fibrogenesis, a process that involves several cell types including KCs, HSCs and recruited leukocytes (Bataller and Brenner, 2005; Friedman, 2008; Pinzani and Macias-Barragan, 2010; Hernandez-Gea and Friedman, 2011). KCs can be activated upon liver injury by phagocytosis of apoptotic bodies (Canbay et al., 2003a). Analyses of frozen sections from wild-type and *Ncu-g1^gt/gt^* livers showed increased staining for CD68, a marker for activated KCs (Kinoshita et al., 2010), in *Ncu-g1^gt/gt^* livers (Fig. 5A). To further characterize the KCs, staining for F4/80 revealed swollen and hypertrophic macrophages (Fig. 5B), displaying a post-phagocytic phenotype. Because inflammatory macrophages have been shown to switch to a restorative subset after phagocytosis (Ramachandran et al., 2012), we investigated the expression of an inflammatory (*Cd86*) and a restorative (*Cd81*) marker (Ramachandran et al., 2012) by qPCR. Fig. 5C shows a significant increase in *Cd86* expression, suggesting that the activated macrophages in *Ncu-g1^gt/gt^* livers belong to a pro-inflammatory subtype (Ramachandran et al., 2012).

Activated KCs stimulate HSC activation by secreting reactive oxygen species and pro-fibrotic cytokines, such as TNF-α (Canbay et al., 2003a), TGF-β and PDGF (Bataller and Brenner, 2005; Friedman, 2008). Fig. 5D,E show increased expression of mRNA for all three cytokines in addition to a receptor for TGF-β, thus confirming activation of KCs. When HSCs are activated by pro-fibrotic cytokines, they evolve into myofibroblast-like cells and start expressing α-Sma, Vim, Igfbp3 and collagen (predominantly type I), characteristic for their activation (Boers et al., 2006; Friedman, 2008). This was demonstrated by the increased expression of *α-Sma, Igfbp3* and *Vim* as shown in Fig. 5F and supported by immunostaining of liver sections from *Ncu-g1^gt/gt^* mice, showing the presence of star-shaped α-Sma-positive cells in the liver parenchyma (Fig. 5G). Taken together, these results point to activation of both KCs and HSCs in *Ncu-g1^gt/gt^* liver. When activated, these cells recruit white blood cells to sites of lesion, depicted in Fig. 3A and Fig. 4A (Maher, 2001; Bataller and Brenner, 2005; Zhan et al., 2006). The qPCR arrays suggested a massive increase in the mRNA levels for myeloperoxidase. This was confirmed at the protein level by immunofluorescence (Fig. 5H) and verified by qPCR (Fig. 5I). Myeloperoxidase is most probably produced by the infiltrating polymorphonuclear leukocytes (Ford, 2010; Amanzada et al., 2011). This enzyme was found in a clustered distribution in small cells, and showed a low degree of colocalization with KCs, another cell type capable of producing this enzyme (Brown et al., 2001) (supplementary material Fig. S1). In support of these findings, the mRNA level of S100 calcium binding protein A8 (S100A8), a protein known to be involved in chemotaxis of polymorphonuclear leukocytes (Ryckman et al., 2003), was significantly upregulated in *Ncu-g1^gt/gt^* liver (Fig. 5J).

### Accumulation of autofluorescent material in *Ncu-g1^gt/gt^* Kupffer cells

**Fig. 6.**
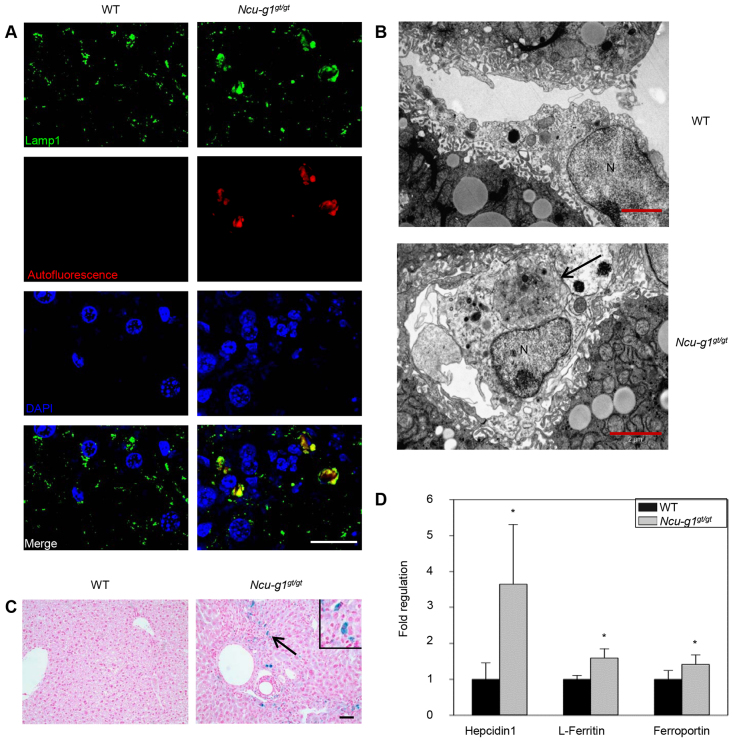
**Intralysosomal accumulation of lipofuscin and iron in *Ncu-g1^gt/gt^* KCs.** (A) Immunofluorescence image analyses showed colocalization of Lamp1-coated vesicles (green) and autofluorescence (red) in *Ncu-g1^gt/gt^* but not in wild-type (WT) liver. Liver sections were counterstained with DAPI (blue). Scale bar: 20 μm. (B) Representative transmission electron microscopy micrographs of KCs from WT and *Ncu-g1^gt/gt^* liver. Large storage vacuoles are visible inside *Ncu-g1^gt/gt^* KCs (arrow) (N, nucleus). Scale bars: 2 μm. (C) Perls’ Prussian blue staining (blue) of liver sections from WT and *Ncu-g1^gt/gt^* mice revealed accumulation of iron (arrow, insert) in *Ncu-g1^gt/gt^* KCs. Scale bar: 50 μm. (D) qPCR analyses show elevated expression of genes involved in iron accumulation (*Hepcidin1, L-Ferritin*) and iron transport (*Ferroportin*) in *Ncu-g1^gt/gt^* liver. *n*=4, *P<0.05. Values are expressed as mean±s.e.m.

Dysfunction of lysosomal proteins is often associated with intralysosomal storage of undigested material due to impaired hydrolytic or transport activity (Futerman and van Meer, 2004). Hence, we investigated the possibility of storage in *Ncu-g1^gt/gt^* liver lysosomes. Fig. 6A shows immunofluorescence staining for Lamp1, a marker for lysosomes. Lamp1-positive vesicles were massively enlarged. Fluorescence examination revealed that autofluorescent material was present in these enlarged Lamp1-coated vesicles, and colocalized with CD68-positive staining, indicating activated KCs (supplementary material Fig. S2A). Storage in KCs was confirmed by electron microscopy, where large storage vacuoles were observed in this cell type in *Ncu-g1^gt/gt^* liver (Fig. 6B). Such structures were not obviously seen in other cell types (data not shown). Affected KCs were identified based on morphology after immunoreactivity for F4/80 (supplementary material Fig. S2B). These cells were also positively stained with Perls’ Prussian blue (Fig. 6C and supplementary material Fig. S2B), indicating storage of iron in *Ncu-g1^gt/gt^* liver KCs. Gene expression analyses showed that *Ncu-g1* mRNA was expressed in isolated parenchymal as well as non-parenchymal cells from wild-type liver, ruling out the possibility that storage in KCs, but not hepatocytes, was a result of a cell-type-specific lack of *Ncu-g1* expression (data not shown). In support of these findings, the mRNA levels of Hepcidin1 and L-Ferritin, proteins involved in intracellular iron accumulation (Ganz, 2011), were shown to be elevated in *Ncu-g1^gt/gt^* liver (Fig. 6D). In addition, the expression of the iron transporter Ferroportin was increased (Fig. 6D).

**Fig. 7.**
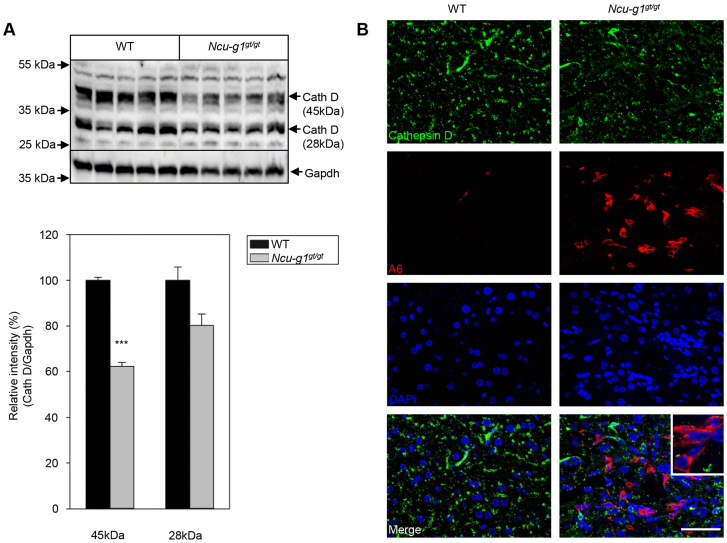
**Reduced expression of cathepsin D in *Ncu-g1^gt/gt^* liver.** (A) Western blot analyses of whole liver homogenates show a significantly reduced protein expression of cathepsin D in *Ncu-g1^gt/gt^* liver (*n*=4, ***P<0.001). Values are expressed as mean±s.e.m. (B) Immunofluorescence image analyzing colocalization of cathepsin D (green) and the oval cell marker A6 (red). Liver sections were counterstained with DAPI (blue). Cathepsin D does not appear to colocalize with A6-positive cells in *Ncu-g1^gt/gt^* liver (inset). Scale bar: 50 μm.

Next, we investigated the expression and activity of a selection of lysosomal enzymes known to be causative for lysosomal disorders. Immunofluorescence studies of cathepsin D expression showed colocalization with CD68 in liver sections from wild-type and *Ncu-g1^gt/gt^* mice (supplementary material Fig. S3). However, the overall expression of cathepsin D was reduced in *Ncu-g1^gt/gt^* liver (supplementary material Fig. S3). This finding was confirmed by western blot analyses where the expression of full-length cathepsin D was significantly reduced (Fig. 7A). Cathepsin D reduction has been shown during the process of tissue regeneration (Suleiman et al., 1980; Fernández et al., 2004). Chronic liver injury leads to activation of oval cells after hepatocytes have exhausted their proliferative capacity (Conigliaro et al., 2010), and might contribute to the observed increase in Mmp2 and Mmp9 expression as shown in Fig. 3G (Pham Van et al., 2008). We examined the oval cell compartment by immunofluorescence labeling for the specific marker, A6 (Engelhardt et al., 1993). Supplementary material Fig. S4 shows strongly increased staining for A6 in *Ncu-g1^gt/gt^* liver. Further fluorescence studies indicated that the reduced levels of cathepsin D in *Ncu-g1^gt/gt^* liver are partially due to the increased number of oval cells, which do not express cathepsin D (Fig. 7B).

No alterations of other lysosomal enzymes included in this study were detected (supplementary material Fig. S5).

## DISCUSSION

In this study, we have shown that *Ncu-g1^gt/gt^* mice were viable and indistinguishable from wild-type siblings with regard to growth and fertility. However, lack of NCU-G1 expression led to spontaneous development of liver fibrosis within 6 months. The *Ncu-g1^gt/gt^* mice showed elevated serum ALT and AST activity levels, increased deposition of extracellular collagen, activated KCs and HSCs, infiltrating leukocytes and apoptotic cells. In support of these findings, the expression of marker genes for oxidative stress and fibrosis were increased in the *Ncu-g1^gt/gt^* liver. These phenotypic findings are all key elements in liver fibrogenesis (Bataller and Brenner, 2005; Friedman, 2008; Kisseleva and Brenner, 2008). Furthermore, autofluorescent lipofuscin and iron accumulated in *Ncu-g1^gt/gt^* liver KCs. Despite ubiquitous expression of *Ncu-g1* (Steffensen et al., 2007; Schieweck et al., 2009) and the verified loss of transcript in all assayed organs, this study suggested that lack of NCU-G1 predominantly affects the liver. The observed splenomegaly is likely a secondary effect due to portal vein hypertension.

Lysosomes are present in nearly every cell of the body, but pathological manifestations of lysosomal disorders can be restricted to specific organs as shown for mice with disrupted lysosomal integral membrane protein 1 (Limp1). These animals are both fertile and viable and do not present any overt phenotype, but they suffer from a disturbed water balance, and hence a kidney pathology (Schröder et al., 2009). Limp1 is ubiquitously expressed, so the kidney pathology could be a result of an organ-specific function of Limp1 that cannot be compensated by other proteins (Schröder et al., 2009).

The majority of lysosomal disorders are characterized by intralysosomal accumulation of unmetabolized substrates; however, secondary alterations in other biochemical and cellular pathways contribute to a wide range of clinical symptoms (Futerman and van Meer, 2004). These vary from neurological and developmental problems to internal organ injury (Jevon and Dimmick, 1998; Futerman and van Meer, 2004; Parkinson-Lawrence et al., 2010). Liver fibrosis is a common finding in several lysosomal storage diseases, including Gaucher disease (James et al., 1981; Lachmann et al., 2000), cholesteryl ester storage disease (Desai et al., 1987; Bernstein et al., 2013), and Niemann Pick A/B and C disease (Kelly et al., 1993; Takahashi et al., 1997). Recent work by Moles et al. demonstrated a direct link between lysosomal dysfunction and liver fibrogenesis through the lysosomal enzyme acidic sphingomyelinase, deficient in Niemann Pick type A/B (Takahashi et al., 1997). This enzyme regulates fibrogenesis through control of HSC activation by the lysosomal proteases cathepsins B and D (Moles et al., 2009; Moles et al., 2010; Moles et al., 2012). Lysosomal cathepsins play a pivotal role in the execution of lysosomal cell death (reviewed in detail in Aits and Jäättelä, 2013). A direct link between hepatocyte cell death and cathepsin B and D leakage into the cytosol is well established (Roberg et al., 1999; Guicciardi et al., 2000; Kågedal et al., 2001; Canbay et al., 2003b). However, in *Ncu-g1^gt/gt^* liver the protein levels of cathepsin B and D are not elevated, indicating that the increased level of cell death and liver fibrosis observed in *Ncu-g1^gt/gt^* mice is not a result of an increased expression of these proteases. Decreased expression of cathepsin D has been reported for regenerating liver after partial hepatectomy, where the hepatocytes proliferate and restore liver volume within ~10 days (Suleiman et al., 1980; Fernández et al., 2004). In *Ncu-g1^gt/gt^* liver the reduced level of cathepsin D might result from the observed lack of cathepsin D expression in oval cells. Oval cells contribute to liver regeneration when the proliferative capacity of hepatocytes is exhausted or compromised (Conigliaro et al., 2010), as often seen in conjunction with chronic liver injury (Roskams et al., 2003; Conigliaro et al., 2010), and their activation has been shown to be closely related to inflammation and cytokine production (Knight et al., 2005). Activation of the oval cell compartment, as seen in the *Ncu-g1^gt/gt^* liver, indicates a suppressed hepatocyte proliferation at 6 months of age. The transgenic insults in the *Ncu-g1^gt/gt^* mouse are chronic and accompanied by massive infiltration of inflammatory cells, increased apoptosis and elevated expression of cytokines, yet these mice maintain their liver:body weight ratio and sufficient liver function to avoid progression to cirrhosis and death.

Spontaneous development of liver fibrosis in combination with a similar longevity as for the *Ncu-g1^gt/gt^* mice has been demonstrated in only a few transgenic mouse models. Among these are the *Taut^−/−^* mouse model and the hepatocyte-specific deletions of *Bcl-xL* or *Mcl-1*, all displaying elevated rates of hepatocyte apoptosis as the fibrogenic inducer (Takehara et al., 2004; Warskulat et al., 2006; Vick et al., 2009; Weber et al., 2010). Interestingly, the liver integrity in the *Taut^−/−^* mouse is maintained by oval cell proliferation (Warskulat et al., 2006). The ability of *Ncu-g1^gt/gt^* mice to maintain sufficient liver function and integrity with older age is currently under investigation.

With regard to putatively affected pathways in which NCU-G1 might be involved, the detection of autofluorescent lipofuscin might give some clues. Intralysosomal accumulation of unmetabolized substrates is often caused by deficiencies of soluble enzymes, but some are caused by dysfunction or absence of transmembrane proteins, some of which act as exporters of metabolites through the lysosomal membrane (Futerman and van Meer, 2004). Whether NCU-G1 with its single transmembrane region (Sardiello et al., 2009; Schieweck et al., 2009; Schröder et al., 2010) might serve any export function similar to the Lamp2-mediated cholesterol export from lysosomes remains to be determined (Schneede et al., 2011). Lipofuscin is an intralysosomal polymeric material, undegradable by lysosomal enzymes. It originates from oxidative damage to macromolecules, often associated with degradation of iron-containing proteins (Terman and Brunk, 2004). KCs contribute to the iron metabolism through phagocytosis of erythrocytes (Graham et al., 2007) and excess storage of heme in KCs has been shown to promote oxidative stress, increase hepatic inflammation, induce hepatocyte apoptosis and stimulate fibrogenesis (Otogawa et al., 2007). In support of the hypothesis that storage material in phagolysosomes of KCs can be causative for the inflammation observed in *Ncu-g1^gt/gt^* liver, the threefold increase of CD86 expression suggests that the *Ncu-g1^gt/gt^* hepatic macrophage subset is pro-inflammatory (Ramachandran et al., 2012). However, this result needs to be interpreted with caution, because liver sinusoidal endothelial cells are also known to express this marker (Knolle et al., 1998; Knolle and Limmer, 2003).

*Ncu-g1^gt/gt^* KCs also show accumulation of iron, as detected by Perls’ Prussian blue staining, coinciding with an elevated hepatic expression of the iron storage protein L-Ferritin (Recalcati et al., 2008). The plasma membrane iron exporter, Ferroportin, is expressed exclusively by KCs in liver (Knutson et al., 2003), and its expression is induced by erythrophagocytosis and iron loading (Knutson et al., 2003). During a state of inflammation, elevated Hepcidin1 levels target Ferroportin for degradation (Collins et al., 2008; Ganz, 2011). In agreement with the observed iron accumulation in KCs, the expression of Hepcidin1 and Ferroportin was increased in *Ncu-g1^gt/gt^* liver. These results also suggest a possible accumulation of erythrocyte-derived storage material in the phagolysosomes of *Ncu-g1^gt/gt^* KCs. An essential heme transporter from the phagolysosomes of macrophages (HRG1) has recently been described (White et al., 2013). Whether the glycosylated luminal tail of NCU-G1 might interact directly with HRG1 or offer protection against lysosomal proteases (Schieweck et al., 2009) remains to be elucidated. However, we cannot finally rule out that lipofuscin and iron deposition are rather a consequence than the cause of liver fibrosis.

In summary, we have created a viable *Ncu-g1^gt/gt^* mouse with spontaneous liver fibrosis as the predominant phenotype. The condition progresses slowly, but displays all hallmarks of fibrosis at 6 months of age. Elevated markers for oxidative stress and inflammation were detected in *Ncu-g1^gt/gt^* liver, in addition to increased accumulation of lipofuscin and iron in KCs. Fibrosis has many etiologies and consists of acute as well as chronic liver damage. It is well known that reversal of fibrosis is possible if the cause is remedied; however, that is often not possible. In the constant search for improved drugs to treat this condition, various animal models are essential tools. We propose that the *Ncu-g1^gt/gt^* mouse could be useful in the development of treatments for attenuation of fibrosis due to chronic liver damage.

## MATERIALS AND METHODS

### Generation of *Ncu-g1^gt/gt^* mice

Embryonic stem cells (ES cells 129S2, P084H04) carrying a gene-trap in the first intron of the *Ncu-g1* gene (RIKEN cDNA 0610031J06 gene) were purchased from The German Gene-Trap Consortium (Neuherberg, Germany). The gene-trap leads to a stop in transcription at this point. In collaboration with the Norwegian Transgenic Centre (Oslo, Norway), ES cells were injected into C57BL/6 blastocysts and chimeric mice were obtained. Five chimeric males were mated with C57BL/6 females. The first litters consisted of 29 pups out of which nine were positive for neomycin and seven of these were shown to carry the gene-trap by PCR. Two females and one male were chosen for further breeding and pups were analyzed for presence of the gene-trap by PCR, using 200 ng of ear genomic DNA and the primer pairs shown in supplementary material Table S2 to differentiate between wild-type and gene-trap-carrying mice: *FlpROSA*, yielding a product of 811 bp when the gene-trap cassette is present; and *NCUG1*, yielding a 562 bp product when the gene-trap cassette is absent. Founders were selected and mated to yield the F1 generation. Mating resulted in mice heterozygous for the *Ncu-g1* gene modification. Heterozygotes were mated, leading to the birth of homozygotes. Mice were maintained in an approved animal facility (National Lab Animal Center, The Norwegian Institute of Public Health, Oslo, Norway) with access to food and water *ad libitum*. Animal handling was according to national laws and regulations.

### Analysis of gene expression

RNA extractions from mouse liver, kidney, lung and spleen (*n*=3) were carried out according to the manufacturer using RNeasy Plus kit from Qiagen (Hilden, Germany). Expression of *Ncu-g1* mRNA was assessed using TaqMan gene expression assays for detection of mouse *Ncu-g1*: Mm00658309_g1, and mouse *Gapdh*: Mm99999915_g1 (Life Technologies, Carlsbad, CA, USA), and LightCycler^®^ 480 Probes Master (Roche Applied Science, Mannheim, Germany). Signals below the threshold value (cp >40) were manually set to 40 in the calculations. RNA was extracted from livers of wild-type and *Ncu-g1^gt/gt^* mice (*n*=3), and gene expression was analyzed using the Fibrosis (PAMM-120A, Qiagen), Hepatotoxicity (PAMM-093F, Qiagen), Signal Transduction Pathway Finder (PAMM-014A, Qiagen) and Oxidative Stress and Antioxidant Defense RT^2^ Profiler™ Array (PAMM-065A, Qiagen), following the manufacturer’s instructions. To verify array results, and to further elucidate the fibrogenic response, RNA from livers of wild-type and *Ncu-g1^gt/gt^* mice (*n*=4, age 6 months) was analyzed for relative gene expression of selected mRNA transcripts by qPCR (supplementary material Table S3). Analysis was performed using LightCycler^®^ 480 SYBR Green I Master Mix (Roche Applied Science). Relative gene expression was calculated using β-actin and eukaryotic translation elongation factor 2 as reference genes.

Isolation of mouse liver cells from wild-type mice was carried out by the two-step perfusion method as described (Hansen et al., 2002). Liver parenchymal and non-parenchymal cells were separated by differential centrifugation and Pronase E (Merck, Darmstadt, Germany) treatment as described elsewhere (Berg and Boman, 1973). The expression of *Ncu-g1* was analyzed as described above.

### Histochemistry

Livers from 6-month-old *Ncu-g1^gt/gt^* (*n*=6) and wild-type (*n*=3) mice were collected and fixed in 4% formaldehyde in 0.1 M PBS. Representative tissue blocks (5 mm thick) were embedded in paraffin, sliced in 4-μm sections and stained with H&E, Masson-Goldner’s trichrome staining for collagen, or Perls’ Prussian blue staining for iron according to standard procedures.

### Immunohistochemistry

For labeling of α-Sma and active caspase 3, formalin-fixed paraffin-embedded sections were deparaffinized, rehydrated and demasked in a microwave oven for 24 minutes in Tris/EDTA buffer (pH 9.1). Rabbit polyclonal anti-α-Sma (1:500, ab5694, Abcam, Cambridge, MA, USA) and rabbit polyclonal anti-active caspase 3 (1:500, G7481, Promega, Madison, WI, USA) were used as antibodies. For labeling of F4/80, formalin-fixed paraffin-embedded sections were deparaffinized, rehydrated and demasked in a microwave oven for 24 minutes in Target retrieval solution (pH 6.0–6.2). Rat monoclonal anti-F4/80 (1:100, 14-4801, eBioscience, San Diego, CA, USA) and peroxidase-conjugated AffiniPure F(ab′)2 fragment mouse anti-rat IgG (H^+^L) (1:100, 212-036-168, Jackson ImmunoResearch Europe Ltd, Newmarket, Suffolk, UK) were used as primary and secondary antibodies, respectively.

The antigen-antibody complexes were visualized with Dako Cytomation Envision+ System-HRP (K4007, DAKO North America Inc., CA, USA) using 3,3′-diaminobenzidine as the chromogen. The sections were counterstained with Mayer’s hematoxylin solution.

### Immunofluorescence

Formalin-fixed livers were sectioned using a Leica 9000s sliding microtome (Wetzlar, Germany) into 40-μm-thick free-floating sections. After blocking with 4% normal goat serum and permeabilization with 0.5% Triton X-100 in 0.1 M phosphate buffer (PB), sections were incubated with appropriate primary antibodies overnight. Antibodies used for immunofluorescence were: rat monoclonal anti-CD68 (1:500, FA-11, AbD Serotec, Oxford, UK); rabbit polyclonal anti-myeloperoxidase (1:300, Millipore, Billerica, MA, USA); rat monoclonal anti-F4/80 (1:25 cell culture supernatant). The monoclonal antibody against mouse Lamp1 (clone 1D4B) was obtained from the Developmental Studies Hybridoma Bank developed under the auspices of the NICHD and maintained by The University of Iowa, Department of Biology, Iowa City, IA 52242. Rat monoclonal anti-A6 (cell culture supernatant; 1:25) was a kind gift from Valentina Factor and described previously (Engelhardt et al., 1990). Cathepsin D antibody was described previously (Claussen et al., 1997). After washing with 0.25% Triton X-100 in PB and incubation with Alexa-Fluor-488 or -633 secondary antibodies (Molecular Probes, Eugene, OR, USA), sections were counterstained with DAPI and mounted with Mowiol/DABCO. Confocal laser scanning microscopy was performed on a Leica TCS SP2 microscope (Wetzlar, Germany).

### Transmission electron microscopy (TEM)

*Ncu-g1^gt/gt^* and wild-type mice (*n*=3, age 6 months) were subjected to perfusion fixation using HEPES buffer (0.1 M, pH 7.2–7.4) with 4% formaldehyde and 2.5% glutaraldehyde. Livers were cut into tissue blocks of 1 mm^3^, transferred to new fixative solution and kept at 4°C overnight. The samples were rinsed 2×10 minutes in 0.1 M sodium cacodylate buffer prior to post-fixation (2% OsO_4_ in 0.1 M sodium cacodylate buffer) for 1 hour, and rinsed 5×10 minutes in distilled water before bulk staining with 1.5% uranyl acetate [(CH_3_COO)_2_UO_2_·2H_2_O] in distilled water for 30 minutes in the dark. Fixed tissue samples were dehydrated in a graded series of ethanol for 10 minutes each (70%, 80%, 90%, 96%), 4×15 minutes in 100% ethanol and finally 2×10 minutes in propylene oxide. Following dehydration, tissue samples were infiltrated with epoxy:propylene oxide (1:1) for 30–60 minutes on a rotary shaker, evaporated overnight, and the following day incubated 1 hour in pure epoxy before embedding in plastic capsules and polymerization at 60°C. Five tissue blocks were selected per individual mouse. Ultrathin sections were obtained using a Leica Ultracut S microtome (Leica, Wetzlar, Germany) with diamond knife. The sections were collected on copper-coated grids and stained with lead citrate for 20 seconds. All electron micrographs were obtained with a CM200 transmission microscope (Philips, Amsterdam, The Netherlands) at 80 kV and at least 20 unique micrographs were analyzed in order to produce representative figures.

### Sirius-red quantification of total liver collagen

Quantification of total collagen content from mouse liver tissue was carried out by a sirius-red colorimetric plate assay as described (Kliment et al., 2011). Briefly, livers were homogenized in 10 volumes of CHAPS buffer (50 mM Tris-HCl, pH 7.4, containing 150 mM NaCl, 10 mM CHAPS and protease inhibitors) using an Ultra-Turrax. The samples were diluted using PBS, dried at 37°C overnight on a microtiter plate, and total liver collagen content was determined colorimetrically using 0.1% sirius-red stain (Direct red 80, Sigma-Aldrich, St Louis, MO, USA) in saturated picric acid, and rat tail collagen (Sigma-Aldrich) as standards.

### Western blotting

Liver tritosomes were isolated as described elsewhere (Schieweck et al., 2009). Tritosome preparations (20 μg protein) were electrophoresed on NuPAGE^®^ 4–12% Bis-Tris Mini Gels (Life Technologies), and transferred onto PVDF membranes (Bio-Rad, Hercules, CA, USA). After blocking, membranes were incubated overnight at 4°C with rabbit anti-NCU-G1 serum (Schieweck et al., 2009) or rabbit anti-Lamp1 (1:1000, C54H11, Cell Signaling, Beverly, MA, USA). This was followed by 1 hour incubation with goat anti-rabbit secondary antibody conjugated to horseradish peroxidase (1:4000, 65-6120, Life Technologies). For liver homogenates, livers were homogenized in 20 volumes of lysis buffer (tris-buffered saline containing 1% Triton X-100 and protease inhibitors) by Potter Elvehjem homogenizer, incubated on ice for 30 minutes with continuous vortexing and cleared by centrifugation for 20 minutes at 14,000 ***g***. The supernatants were used for western blotting after separation on 15% SDS-PAGE gels and transfer onto PVDF membranes (Bio-Rad). After incubation with primary antibodies overnight [caspase 3: 1:1000, Cell Signaling, Beverly, MA, USA; cathepsin D: 1:1000 (Claussen et al., 1997); cathepsin B and Gapdh: 1:300 each, Santa Cruz Antibodies] and washing, membranes were incubated for 1 hour with goat anti-rabbit secondary antibody conjugated to horseradish peroxidase (1:5000, Dianova). Protein bands were visualized using Amersham ECL Plus Western Blotting Detection Reagents (GE Healthcare, Pittsburgh, PA, USA).

### Enzyme activity assays

Enzymatic activities of lysosomal hydrolases were determined from liver homogenates (see above) using standard procedures with colorimetric substrates (p-Nitrophenyl-α-D-mannopyranoside for α-mannosidase and p-Nitrophenyl N-acetyl-β-D-glucosaminide for β-hexosaminidase). Absorption of nitrophenolate was determined at 405 nm in a 96-well plate reader. For cathepsin B determination, protease inhibitors were omitted from the lysis buffer. Cathepsin B was determined using fluorescence substrate Z-Arg-Arg-AMC (Bachem, Weil am Rhein, Germany) according to Barrett (Barrett, 1980).

### Serum analysis

Coagulated blood samples from wild-type and *Ncu-g1^gt/gt^* mice (*n*=4) were centrifuged at 1500 ***g*** for 20 minutes, and serum ALT and AST activities were measured at The Central Laboratory, Department of Basic Sciences and Aquatic Medicine, Norwegian School of Veterinary Science.

### Statistical methods

All results are expressed as mean±s.e.m. Genotype distribution was analyzed using the Chi-square test (Microsoft Excel, Microsoft Corporation, Redmond, WA, USA). Other data were analyzed using two-tailed *t*-test (SigmaPlot™, Systat Software Inc., Chicago, IL, USA).

## Supplementary Material

Supplementary Material
