# Supplementary material for: Loss of lysosomal membrane protein NCU-G1 in mice results in spontaneous liver fibrosis with accumulation of lipofuscin and iron in Kupffer cells
Source: Dis Model Mech. 2014 Jan 30;7(3):351–62. doi: 10.1242/dmm.014050 (PMC3944495; doi:10.1242/dmm.014050)
Supplement: Supplementary Material [file supp_7_3_351__index.html]

Loss of lysosomal membrane protein NCU-G1 in mice results in spontaneous liver fibrosis with accumulation of lipofuscin and iron in Kupffer cells — Supplementary Material 

# Loss of lysosomal membrane protein NCU-G1 in mice results in spontaneous liver fibrosis with accumulation of lipofuscin and iron in Kupffer cells

## DMM014050 Supplementary Material

**Files in this Data Supplement:**

- **Supplementary Material**
