# Supplementary material for: Loss of lysosomal membrane protein NCU-G1 in mice results in spontaneous liver fibrosis with accumulation of lipofuscin and iron in Kupffer cells
Source: Dis Model Mech. 2014 Jan 30;7(3):351–62. doi: 10.1242/dmm.014050 (PMC3944495; doi:10.1242/dmm.014050)
Supplement: Supplementary Material [file supp_7.3.351_DMM014050.pdf]

SUPPLEMENTAL TABLES

Table S1. Fold regulation in genes up- or down-regulated more than 1.5-fold in 6 months old *NCU-G1*$^{gt/gt}$ liver vs. wild type liver.

Oxidative stress

Gene symbol	Fold regulation	Description	GeneBank
Mpo	59.6	Myeloperoxidase	NM_010824
Cygb	4.0	Cytoglobin	NM_030206
Nos2	3.9	Nitric oxide synthase 2, inducible	NM_010927
Gpx3	3.8	Glutathione peroxidase 3	NM_008161
Ehd2	3.6	EH-domain containing 2	NM_153068
Slc38a1	3.4	Solute carrier family 38, member 1	NM_134086
Ncf2	3.3	Neutrophil cytosolic factor 2	NM_010877
Cyba	3.0	Cytochrome b-245, alpha polypeptide	NM_007806
Gpx6	3.0	Glutathione peroxidase 6	NM_145451
Txnrd2	2.9	Thioredoxin reductase 2	NM_013711
Epx	2.6	Eosinophil peroxidase	NM_007946
Prnp	2.5	Prion protein	NM_011170
Vim	2.3	Vimentin	NM_011701
Fmo2	2.0	Flavin containing monooxygenase 2	NM_018881
Sod3	1.9	Superoxide dismutase 3, extracellular	NM_011435
Ptgs1	1.8	Prostaglandin-endoperoxide synthase 1	NM_008969
Als2	1.7	Amyotrophic lateral sclerosis 2 (juvenile) homolog (human)	NM_028717
Fancc	1.6	Fanconi anemia, complementation group C	NM_007985
Xirp1	1.6	Xin actin-binding repeat containing 1	NM_011724
Gpx8	1.6	Glutathione peroxidase 8 (putative)	NM_027127
Noxa1	1.6	NADPH oxidase activator 1	NM_172204
Scd1	-1.8	Stearoyl-Coenzyme A desaturase 1	NM_009127

Fibrosis

Gene symbol	Fold regulation	Description	GeneBank
Plau	11.4	Plasminogen activator, urokinase	NM_008873
Thbs1	9.2	Thrombospondin 1	NM_011580
Mmp8	8.8	Matrix metallopeptidase 8	NM_008611
Cxcr4	8.2	Chemokine (C-X-C motif) receptor 4	NM_009911
Mmp13	7.6	Matrix metallopeptidase 13	NM_008607
Pdgfb	7.1	Platelet derived growth factor, B polypeptide	NM_011057
Plat	5.5	Plasminogen activator, tissue	NM_008872
Il10	4.7	Interleukin 10	NM_010548
Serpine1	3.6	Serine (or cysteine) peptidase inhibitor, clade E, member 1	NM_008871
Cav1	3.6	Caveolin 1, caveolae protein	NM_007616
Itgb3	3.2	Integrin beta 3	NM_016780
Thbs2	3.0	Thrombospondin 2	NM_011581
Ccr2	2.9	Chemokine (C-C motif) receptor 2	NM_009915
Fasl	2.8	Fas ligand (TNF superfamily, member 6)	NM_010177
Tgfb1	2.6	Transforming growth factor, beta 1	NM_011577
Col1a2	2.6	Collagen, type I, alpha 2	NM_007743
Ccl3	2.5	Chemokine (C-C motif) ligand 3	NM_011337
Il1b	2.4	Interleukin 1 beta	NM_008361
Egf	2.3	Epidermal growth factor	NM_010113
Mmp9	2.3	Matrix metallopeptidase 9	NM_013599
Timp2	2.3	Tissue inhibitor of metalloproteinase 2	NM_011594
Serpinh1	2.2	Serine (or cysteine) peptidase inhibitor, clade H, member 1	NM_009825
Ltbp1	2.0	Latent transforming growth factor beta binding protein 1	NM_019919
Tgfb3	2.0	Transforming growth factor, beta 3	NM_009368
Stat1	1.8	Signal transducer and activator of transcription 1	NM_009283
Smad6	1.8	MAD homolog 6 (Drosophila)	NM_008542
Nfkb1	1.7	Nuclear factor of kappa light polypeptide gene enhancer in B-cells 1, p105	NM_008689
Ccl12	1.7	Chemokine (C-C motif) ligand 12	NM_011331
Col3a1	1.6	Collagen, type III, alpha 1	NM_009930
Il1a	1.6	Interleukin 1 alpha	NM_010554
Akt1	1.6	Thymoma viral proto-oncogene 1	NM_009652
Itga1	1.5	Integrin alpha 1	NM_001033228
Dcn	1.5	Decorin	NM_007833
Mmp14	1.5	Matrix metallopeptidase 14 (membrane-inserted)	NM_008608
Tgif1	-1.7	TGFB-induced factor homeobox 1	NM_009372
Stat6	-1.8	Signal transducer and activator of transcription 6	NM_009284
Inhbe	-2.4	Inhibin beta E	NM_008382
Il13ra2	-3.1	Interleukin 13 receptor, alpha 2	NM_008356
Bmp7	-4.2	Bone morphogenetic protein 7	NM_007557

Hepatotoxicity

Gene symbol	Fold regulation	Description	GeneBank
S100a8	22.5	S100 calcium binding protein A8 (calgranulin A)	NM_013650
Col4a1	4.0	Collagen, type IV, alpha 1	NM_009931
Slc2a3	3.8	Solute carrier family 2 (facilitated glucose transporter), member 3	NM_011401
Thrsp	3.1	Thyroid hormone responsive SPOT14 homolog (Rattus)	NM_009381
Hmox1	2.6	Heme oxygenase (decycling) 1	NM_010442
Icam1	2.2	Intercellular adhesion molecule 1	NM_010493
Btg2	2.2	B-cell translocation gene 2, anti-proliferative	NM_007570
Fasn	2.2	Fatty acid synthase	NM_007988
Osmr	2.1	Oncostatin M receptor	NM_011019
Osta	2.0	Organic solute transporter alpha	NM_145932
Cd36	1.9	CD36 antigen	NM_007643
Hao2	1.9	Hydroxyacid oxidase 2	NM_019545
Tagln	1.8	Transgelin	NM_011526
Tmem2	1.8	Transmembrane protein 2	NM_031997
Casp3	1.8	Caspase 3	NM_009810
Fxc1	1.7	Fractured callus expressed transcript 1	NM_019502
Cd68	1.7	CD68 antigen	NM_009853
Scd1	1.6	Stearoyl-Coenzyme A desaturase 1	NM_009127
Cdkn1a	1.5	Cyclin-dependent kinase inhibitor 1A (P21)	NM_007669
Lpl	1.5	Lipoprotein lipase	NM_008509
Hpn	-1.5	Hepsin	NM_008281
Car3	-1.6	Carbonic anhydrase 3	NM_007606
Gclc	-1.6	Glutamate-cysteine ligase, catalytic subunit	NM_010295
Fam158a	-1.6	Family with sequence similarity 158, member A	NM_033146
Igfals	-1.7	Insulin-like growth factor binding protein, acid labile subunit	NM_008340
Abcc3	-1.7	ATP-binding cassette, sub-family C (CFTR/MRP), member 3	NM_029600
Txnrd1	-1.7	Thioredoxin reductase 1	NM_015762
Dnajb11	-1.8	DnaJ (Hsp40) homolog, subfamily B, member 11	NM_026400
Avpr1a	-1.8	Arginine vasopressin receptor 1A	NM_016847
BC031353	-1.9	CDNA sequence BC031353	NM_153584
Mbl2	-2.0	Mannose-binding lectin (protein C) 2	NM_010776
Wipi1	-2.0	WD repeat domain, phosphoinositide interacting 1	NM_145940
Fabp1	-2.0	Fatty acid binding protein 1, liver	NM_017399
Mlxipl	-2.1	MLX interacting protein-like	NM_021455
Bhmt	-2.3	Betaine-homocysteine methyltransferase	NM_016668
Fmo1	-2.4	Flavin containing monooxygenase 1	NM_010231
Lgr5	-2.8	Leucine rich repeat containing G protein coupled receptor 5	NM_010195

Pathway finder

Gene symbol	Fold regulation	Description	GeneBank
Cdkn2a	20.4	Cyclin-dependent kinase inhibitor 2A	NM_009877
Ccl2	7.6	Chemokine (C-C motif) ligand 2	NM_011333
Cxcl9	4.3	Chemokine (C-X-C motif) ligand 9	NM_008599
Ccnd1	4.1	Cyclin D1	NM_007631
Sele	3.8	Selectin, endothelial cell	NM_011345
Icam1	3.6	Intercellular adhesion molecule 1	NM_010493
Hhip	3.2	Hedgehog-interacting protein	NM_020259
Pmepa1	2.9	Prostate transmembrane protein, androgen induced 1	NM_022995
Tert	2.8	Telomerase reverse transcriptase	NM_009354
Vcam1	2.8	Vascular cell adhesion molecule 1	NM_011693
Gys1	2.8	Glycogen synthase 1, muscle	NM_030678
Foxa2	2.7	Forkhead box A2	NM_010446
Naip1	2.4	NLR family, apoptosis inhibitory protein 1	NM_008670
Bcl2	2.4	B-cell leukemia/lymphoma 2	NM_009741
Hsf1	2.4	Heat shock factor 1	NM_008296
Cdkn2b	2.1	Cyclin-dependent kinase inhibitor 2B (p15, inhibits CDK4)	NM_007670
Il1a	2.0	Interleukin 1 alpha	NM_010554
Cxcl1	2.0	Chemokine (C-X-C motif) ligand 1	NM_008176
Wnt2	2.0	Wingless-related MMTV integration site 2	NM_023653
Hk2	2.0	Hexokinase 2	NM_013820
Bmp4	1.8	Bone morphogenetic protein 4	NM_007554
Cd5	1.7	CD5 antigen	NM_007650
Jun	1.6	Jun oncogene	NM_010591
Igfbp3	1.6	Insulin-like growth factor binding protein 3	NM_008343
Trp53	1.6	Transformation related protein 53	NM_011640
Bax	1.6	Bcl2-associated X protein	NM_007527
Cdkn1b	1.5	Cyclin-dependent kinase inhibitor 1B	NM_009875
Ikbkb	1.5	Inhibitor of kappaB kinase beta	NM_010546
Nfkbia	1.5	Nuclear factor of kappa light polypeptide gene enhancer in B-cells inhibitor, alpha	NM_010907
Fn1	1.4	Fibronectin 1	NM_010233
Il4ra	-1.8	Interleukin 4 receptor, alpha	NM_001008700
Fasn	-1.9	Fatty acid synthase	NM_007988
Gadd45a	-2.2	Growth arrest and DNA-damage-inducible 45 alpha	NM_007836

Table S2. Primers used for genotyping

Primer name	5’-Primer	3’-Primer	Expected product size
FlpROSA	GATGTCAAGAAGAGACGTTGGGTT	GCATCGCATTGTCTGAGTAGGTGT	811bp
NCUG1	GGAGAAGAGACTCGCCAGGTAAGG	GCTGCCCACTGCCCGAATA	562bp

Table S3. Primers used in real-time qRT-PCR

Target gene	Description	5'-Primer	3'-Primer
Vim	Vimentin	CGGAAAGTGGAATCCTTGCA	CAGTGAGGTCAGGCTTGGAAA
Igfbp3	Insulin-like growth factor binding protein 3	TCGGACTCCACGTTCAGAGAT	GCCCCACCTCACAACAAACTT
α-Sma	Alpha-smooth muscle actin	CCACCGCAAATGCTTCTAAGT	GGCAGGAATGATTTGGAAAGG
Tgfb1	Transforming growth factor β1	GCACCATCCATGACATGAACC	AAGTCAATGTACAGCTGCCGC
Tgfbr2	Transforming growth factor β-receptor II	CCCCGGAAACTGATGGATTT	TCCGTGTTGTGGTTGATGTTG
Nfkb1	Nuclear factor of kappa light polypeptide gene enhancer in B-cells 1	AAAATGCCCCACGGTTATGG	GGACGATGCAATGGACTGTCA
Tnf	Tumor necrosis factor	CAAACCCTGGTATGAGCCCAT	ACCCATTCCCTTCACAGAGCA
Pdgfb	Platelet-derived growth factor beta polypeptide	ATTGCACATGATGCACTGCG	TCATTCTGAGCTTCAGGCTGG
Col1a2	Collagen, type I, alpha 2	CCAAGAATTCCGTGTGGAGGT	GGAGGAAACGGCAAAGAGAAA
Mmp2	Matrix metallopeptidase 2	TGATAACCTGGATGCCGTCGT	TGCTTCCAAACTTCACGCTCT
Mmp9	Matrix metallopeptidase 9	CTTTGAGTCCGGCAGACAAT	TTCCAGTACCAACCGTCCTT
Timp1	Tissue inhibitor of metalloproteinases 1	GGCATCCTCTTGTTGCTATCACT	CTTATGACCAGGTCCGAGTTGC
Vcam1	Vascular cell adhesion molecule 1	TTCGGTTGTTCTGACGTGTGC	TTTGGCCCCCTCATTCCTT
Ccl2	Chemokine (C-C motif) ligand 2	AGCAGCAGGTGTCCCAAAGAA	CATTTGGTTCCGATCCAGGTT
S100a8	S100 calcium binding protein A8	CCGTCTTCAAGACATCGTTTGA	GTAGAGGGCATGGTGATTTCCT
Mpo	Myeloperoxidase	TCATGTTCCGCCTGAACAATC	AATGCCACCTTCCAACACG
iNos2	Inducible nitric oxide synthase 2	CGGTGCAGTCTTTTCCTATGG	CATTTCGCTGTCTCCCCAA
Gpx3	Glutathione peroxidase 3	AAGATCCATGACATCCGCTGG	TCTTGACGTTGCTGACTGTGG
Sod3	Superoxide dismutase 3	GGCCCCAGGTTCTGTTTTCAT	GGCCAGTGCATTGTGTTGTGA
Noxa1	NADPH oxidase activator 1	TGTGAAGTGGACGAAGCATGG	AAGCATGGCTTCCACATAGGC
Cd81	CD81	TGCTGTACCTGGAACTGGGAAACA	TTGCTGAAGGGCCTGGTCATAGAA
Cd86	CD86	TTGTGTGTGTTCTGGAAACGGAG	AACTTAGAGGCTGTGTTGCTGGG
Hepcidin1	Hepcidin 1	TGCAGAAGAGAAGGAAGAGAGACA	CACACTGGGAATTGTTACAGCATT
L-ferritin	L-Ferritin	CTACTCCGGATCAGCCATGAC	AAGTTGACCAGGCGGTTCAC
Ferroportin	Ferroportin	CTACCATTAGAAGGATTGACCAGCTA	ACTGGAGAACCAAATGTCATAATCTG
eEF2	Eukaryotic translation elongation factor 2	CCATCGCTGAACGCATCAAG	CAGGCCAGAACCAAAGCCTA
β-actin	Beta-actin	AGCCATGTACGTAGCCATCC	GCTGTGGTGGTGAAGCTGTA

SUPPLEMENTAL FIGURE LEGENDS

Fig. S1. Expression of CD68 and myeloperoxidase. Immunofluorescence image analyzing co-localization of CD68 (red) and myeloperoxidase (green). Liver sections were counterstained with DAPI (blue). CD68-positive KC express only little myeloperoxidase in *Ncu-g1*$^{gt/gt}$ liver (long arrows, inset), whereas the majority of myeloperoxidase is expressed by infiltrating polymorphonuclear leukocytes (short arrows). Scale bar: 50µm.

Fig. S2. Co-localization of CD68 and autofluorescence. (A) Immunofluorescence image analyzing co-localization of CD68 (red) and autofluorescence (green). Liver sections were counterstained with DAPI (blue). Autofluorescent material appears to co-localize with CD68-positive cells in *Ncu-g1*$^{gt/gt}$ liver. Scale bar: 50µm. (B) Morphological comparison between F4/80-positive stained cells (upper panel) and Perls' Prussian blue stained cells (lower panel) in *Ncu-g1*$^{gt/gt}$ liver. Scale bar: 10µm.

Fig. S3. Co-localization of Cathepsin D and CD68.
Immunofluorescence image analyses of co-localization of cathepsin D (green) and CD68 (red). Liver sections were counterstained with DAPI (blue). Scale bar: 50µm.

Fig. S4. Active oval cell compartment in *Ncu-g1*$^{gt/gt}$ liver.
Immunofluorescence image show a massive activation/proliferation of the oval cells in *Ncu-g1*$^{gt/gt}$ liver. Scale bar: 50µm.

Fig. S5. Expression and activity of lysosomal enzymes. (A) Western blot analyses of whole liver homogenates show no difference in expression of cathepsin B between wild type (WT)

and *Ncu-g1*$^{gt/gt}$ liver at the protein level. (B) Enzyme activity assays on whole liver homogenates show comparable levels of enzyme activity of cathepsin B, (C) α-mannosidase and (D) β-hexosaminidase in WT and *Ncu-g1*$^{gt/gt}$ liver.

Fig. S1

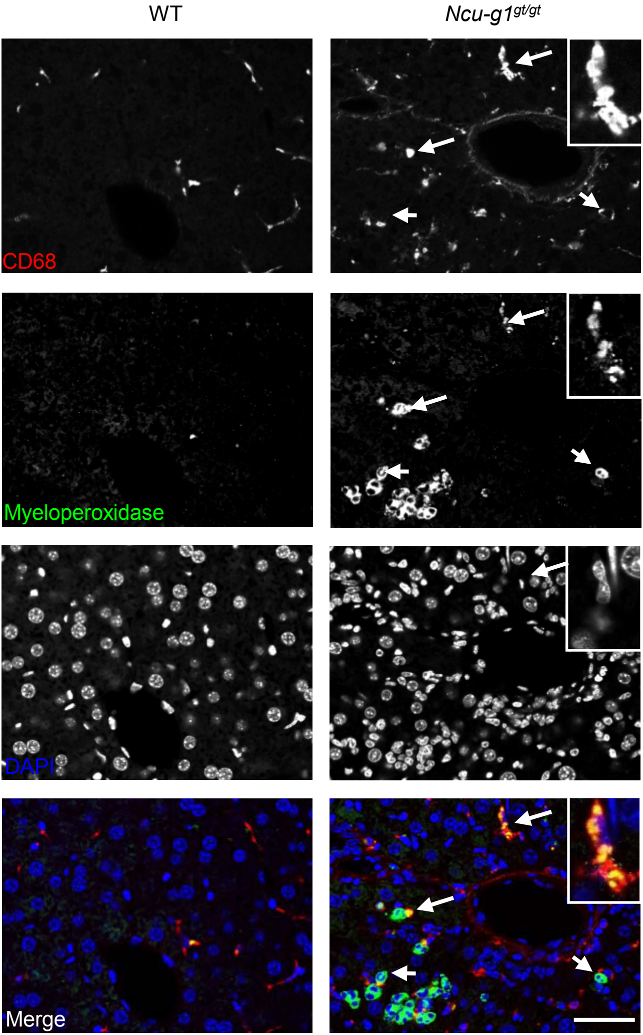

Fig. S2

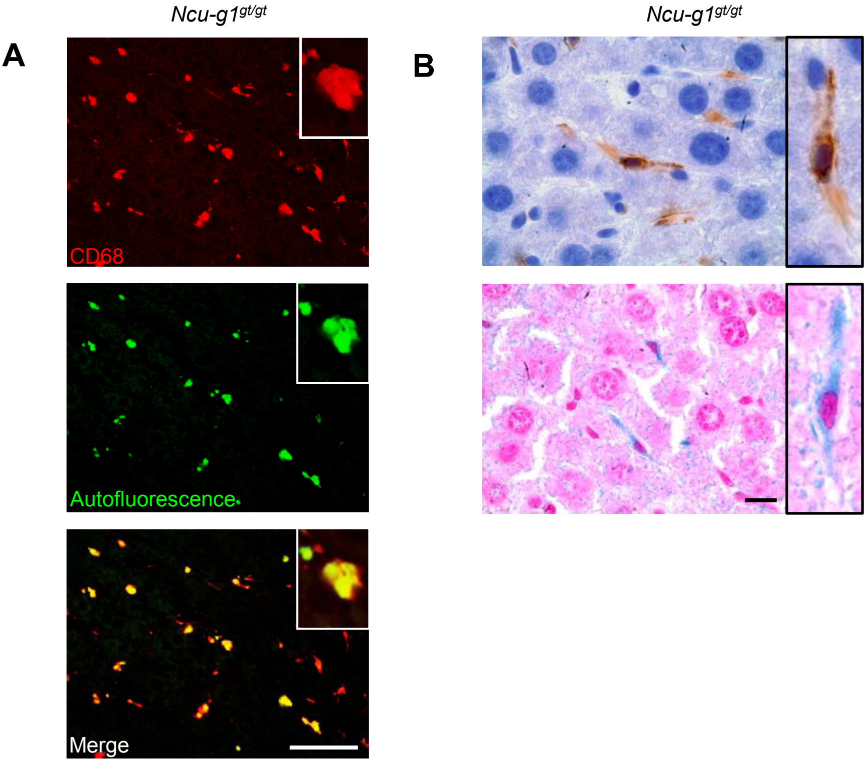

Fig. S3

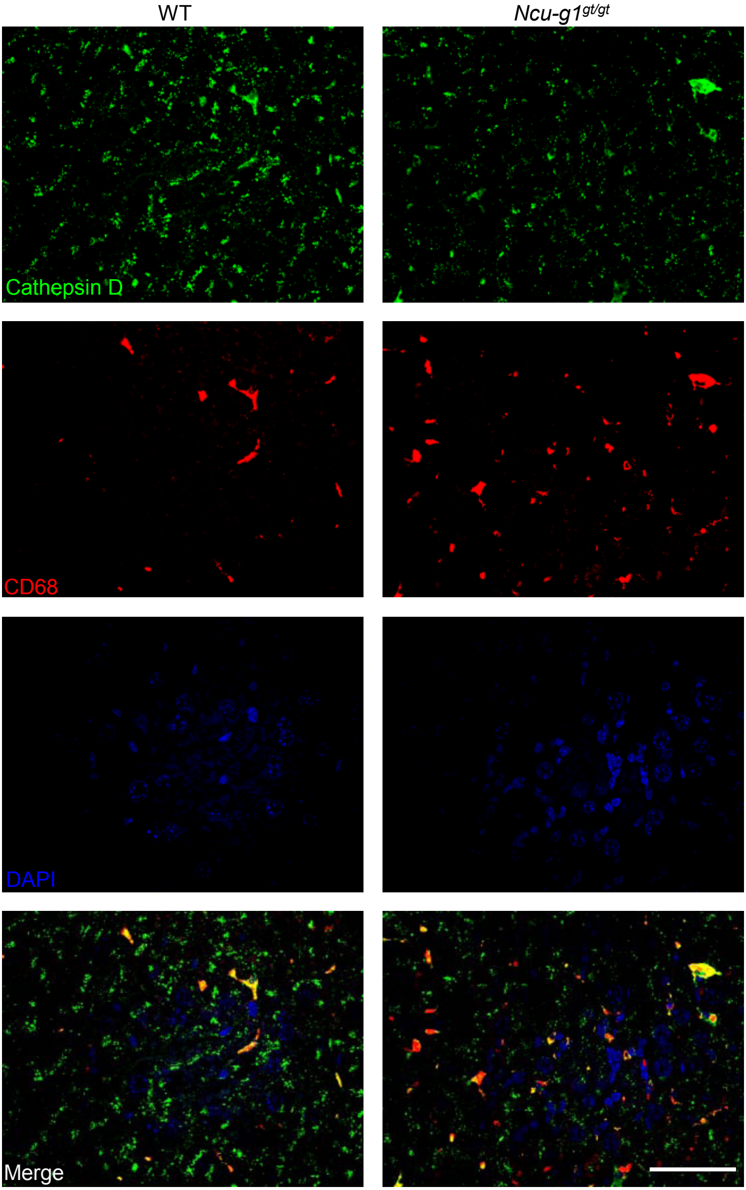

Fig. S4

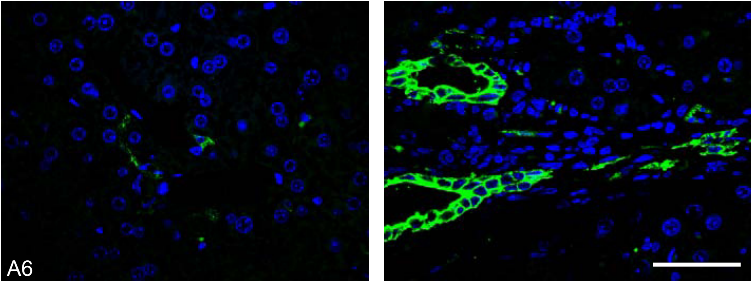

Fig. S5

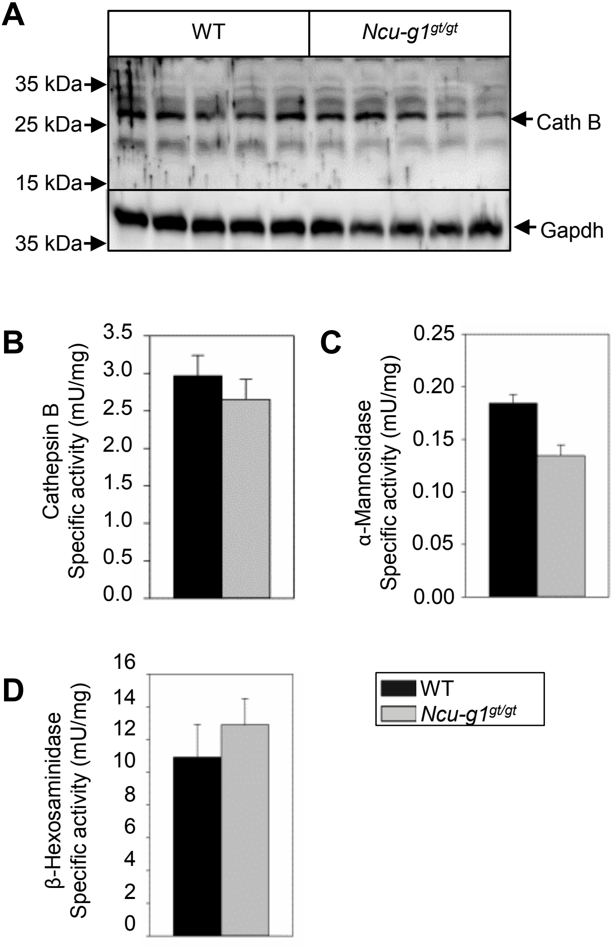

A
WT
Ncu-g1gt/gt
35 kDa
25 kDa
15 kDa
35 kDa
Cath B
Gapdh
B
Cathepsin B
Specific activity (mU/mg)
3.5
3.0
2.5
2.0
1.5
1.0
0.5
0.0
C
α-Mannosidase
Specific activity (mU/mg)
0.25
0.20
0.15
0.10
0.05
0.00
D
β-Hexosaminidase
Specific activity (mU/mg)
16
14
12
10
8
6
4
2
0
WT
Ncu-g1gt/gt
